# Biomedical Applications of Nanotechnology and Nanomaterials

**DOI:** 10.3390/mi8100298

**Published:** 2017-10-02

**Authors:** Vinay Bhardwaj, Ajeet Kaushik

**Affiliations:** 1 Department of Biomedical Engineering, Rutgers-The State University of New Jersey, Piscataway, NJ 08854, USA; 2 Center for Personalized Nanomedicine, Institute of NeuroImmune Pharmacology, Department of Immunology, Herbert Wertheim College of Medicine, Florida International University, Miami, FL 33199, USA

The spurring growth and clinical adoption of nanomaterials and nanotechnology in medicine, i.e. “nanomedicine,” to shape global health care system is a collective effort that comprises academia research, industrial drive, and political and financial support from government. As of today, there are more than 250 nanomedicine products, more than 50 of which are already in the market and being used by doctors or other end-users [1].

The definition and classification of nanomaterials are continuously evolving with our understanding of this exciting field. Adapting from technical and translational information on nanomaterials and nanotechnology from US National Nanotechnology Initiative and European Commission, editors feel it is imperative to mention that nanomaterials’ upper size limit is not restricted to 100 nm [2]. In fact, some commercial nanomedicine products are greater than 100 nm, e.g., abraxane (130 nm) and Myocet (180 nm). Broadly, nanomaterials are categorized as organic, inorganic, or hybrid nanomaterials to highlight their inherent advantages in context to diagnostics and therapeutics. Most, if not all, organic nanomaterials-based medicine carriers use biocompatible polymers and liposomes that are typical carbohydrates, proteins, and lipids found in humans and other animals. The development of new biomaterials and the methods of formulating nanomedicine “intended primarily for therapeutics” in the context of controlled size, stability, percent drug entrapment, and sustained drug release is an always-evolving area of research. Among inorganic nanomaterials, transition metals, including but not limited to gold, silver, platinum, iron, cobalt, titanium, technetium, and lanthanide, have unique optical, electrical, and magnetic properties, which makes them a great choice for multifunctional biomedical applications in optical and electrical sensing [3,4], diagnosis [5,6,7], photo-thermal therapy [8], optogenetics [9], and a few others. In addition, nanomaterials and nanotechnology in conjunction with stem cell biotechnology have great implications in regenerative medicine [10].

Bioactive nanomaterials of polymers and metals are an emerging class of nanomaterials with exciting desired properties. For example, a novel PolymerDrug approach, wherein a polymer is engineered to biodegrade into therapeutically active molecules, such as PolyAspirin, PolyMorphine, and PolyAntibiotics, can improve the therapeutic value of the free form of conventional drugs that are typically prescribed to control pain, inflammation, and infection [11,12]. Another clinically promising nanotechnology approach uses a sugar-based amphiphilic scorpion and star-like nanomaterials with a core-shell micelle design, best suitable geometry for drug encapsulation, and additional properties conferred by their bioactive shells [13]. These bioactive shells have inherent targeting properties that can be tuned for targeted drug delivery to treat cancer, and block scavenger receptors to inhibit artherosclerosis, Parkinson’s, and other diseases with similar pathophysiology [14,15]. In addition to the aforementioned biomedical applications of bioactive polymers, they have implications to engineer biodegradable and bioactive sutures and dressings, drug eluting stents and scaffolds, and medical devices with anti-microbial properties to prevent bio-fouling [16,17,18]. In the last decade or so, we witnessed a spurring growth in biomedical applications of inorganic nanomaterials. In particular, the multifunctional nanotechnology approaches to combine properties of two or more inorganic nanomaterials, i.e. “nanocomposites,” have broadened the horizon of nanotechnology. Nanocomposites are among the best choices for multi-modal imaging to improve diagnosis [19,20] and/or photothermal therapy to compliment chemotherapy [8]. For example, bioactive magneto-electric nanomaterials (MENs) and magneto-optic nanomaterials (MONs) are unique. The magnetic component of these nanomaterials enables magnetically driven targeted drug delivery and magnetic resonance image-guided therapy [21]. An electronic component in these nanocomposites offers actuation properties to remotely control drug release [22,23], and optical components like gold, rare-earth, and quantum dots offer plasmonic, photoluminiscent, and fluorescent properties, respectively. In contrast to polymeric nanomaterials, which are classical drug nanocarriers and best suitable for drug delivery outside the brain space, this special class of ultra-small, magnetically-driven nanocomposites combining electrical (MENs) and optical properties (MONs) are best suitable for brain space [20,21,24,25].

In spite of the significant advancements discussed above, a tunable control over size, stability and functionality of the nanomaterials is required, in particular for their biomedical applications in vivo such as sensing, diagnostics, and therapeutics. The formulation and functionality of novel next-generation nanomaterials should be tuned for maximum practical, “multifunctional,” utility in personalized health care with minimum adverse effects.

The aim of this editorial is to encourage researchers active in this field to submit their manuscript for consideration to publish in this special issue of *Micromachines*. We would like to thank contributors and reviewers for making this special issue a success. I am sure this special issue will be of great interest and value to the scientific community exploring biomedical applications of nanotechnology and nanomaterials.
